# Characteristics of pelvic obliquity in dysplastic hip osteoarthritis

**DOI:** 10.1007/s00402-024-05476-2

**Published:** 2024-08-07

**Authors:** Yuto Ozawa, Yusuke Osawa, Yasuhiko Takegami, Hiroto Funahashi, Shinya Tanaka, Shiro Imagama

**Affiliations:** Department of Orthopaedic Surgery, Nagoya University Graduate School of Medicine, 65 Tsurumai, Showa-ku, Nagoya, 466-8550 Japan

**Keywords:** Pelvic obliquity, Dysplastic hip osteoarthritis, Developmental dysplasia of the hip

## Abstract

**Purpose:**

Factors affecting direction of pelvic obliquity (PO) in dysplastic hip osteoarthritis (DHOA) remains unclear. This retrospective cohort study evaluates morphological characteristics, spinal alignment, and hip function in patients with unilateral DHOA.

**Methods:**

Between 2018 and 2022, 104 patients with unilateral DHA were enrolled. Patients were categorized into flat PO (F-PO group; PO < 2°), affected side PO (A-PO group; PO downward by ≥ 2°), and unaffected side PO (U-PO group; PO upward by ≥ 2°). Demographics, radiographic hip and lower limb parameters, spinal parameters, and functional scores were compared between the groups.

**Results:**

There were 39, 44, and 21 patients in the F-PO, A-PO, and U-PO group, respectively. The subluxation percentage of Crowe classification showed a significant difference among the three groups. The femoral head lateralization distance was significantly greater in the U-PO group than in the F-PO and A-PO groups. Furthermore, the hip adduction angle was significantly lower in the A-PO group than in the F-PO and U-PO groups. The lumbar scoliosis angle was significantly different between the groups. In multivariate analysis, hip adduction angle was extracted as an independent factor associated with the A-PO. Age, subluxation percentage, and hip adduction angle were identified as independent factors associated with the U-PO. Harris hip score was significantly poorer in U-PO group than in F-PO group.

**Conclusions:**

Hip adduction angle influenced A-PO, while age, subluxation percentage, and hip adduction angle influenced U-PO; lumbar scoliosis angle was associated with PO direction. U-PO patients had poorer functional scores, indicating the impact of hip contracture and subluxation on PO direction in DHOA.

## Introduction

Pelvic obliquity (PO) is the lateral inclination of the pelvis in the coronal plane. Spinal deformity, hip joint restrictions, structural changes in pelvic bony components, and leg-length discrepancy have been reported as factors of PO [1]. Dysplastic hip osteoarthritis (DHOA) commonly presents with severe joint deformity and limited range of motion (ROM), and surgical techniques are known to be more difficult than those for general hip osteoarthritis [2]. As subluxation progresses in DHOA, leg-length discrepancies and hip contractures develop. As a result, the progression of subluxation may lead to PO [3, 4]. In addition, there is concern that severe PO may cause adjacent joint disorders. Hence, surgeons treating DHOA should focus on correcting spinal and lower limb alignments, including PO.

Two types of PO are identified in patients who have polio: Type I, characterized by the pelvis tilting downward on the shorter-limb side, and type II, characterized by the pelvis tilting upward on the shorter-limb side [5]. Type I results from shortening of the affected limbs, whereas type II results from external rotator muscle strength and hip adductor contracture as a pain-avoidance mechanism. However, the specific etiology of PO remains unclear [5]. In hip osteoarthritis, PO with the affected side tilts downward (A-PO) is more common [6]. In contrast, in DHOA, both A-PO and PO with the unaffected side tilted downward (U-PO) are observed. However, the relationship between the PO direction and hip morphology, leg length, and spinal alignment in DHOA remains unclear.

In this study, the following four clinical questions were investigated: (1) what are the morphological characteristics of A-PO in DHOA? (2) What are the morphological characteristics of U-PO in DHOA? (3) What factors of spinal alignment are associated with the PO direction in patients who have DHOA? (4) Is there an impact of the PO direction on hip function in DHOA?

## Material and methods

### Patients

**Fig. 1:**
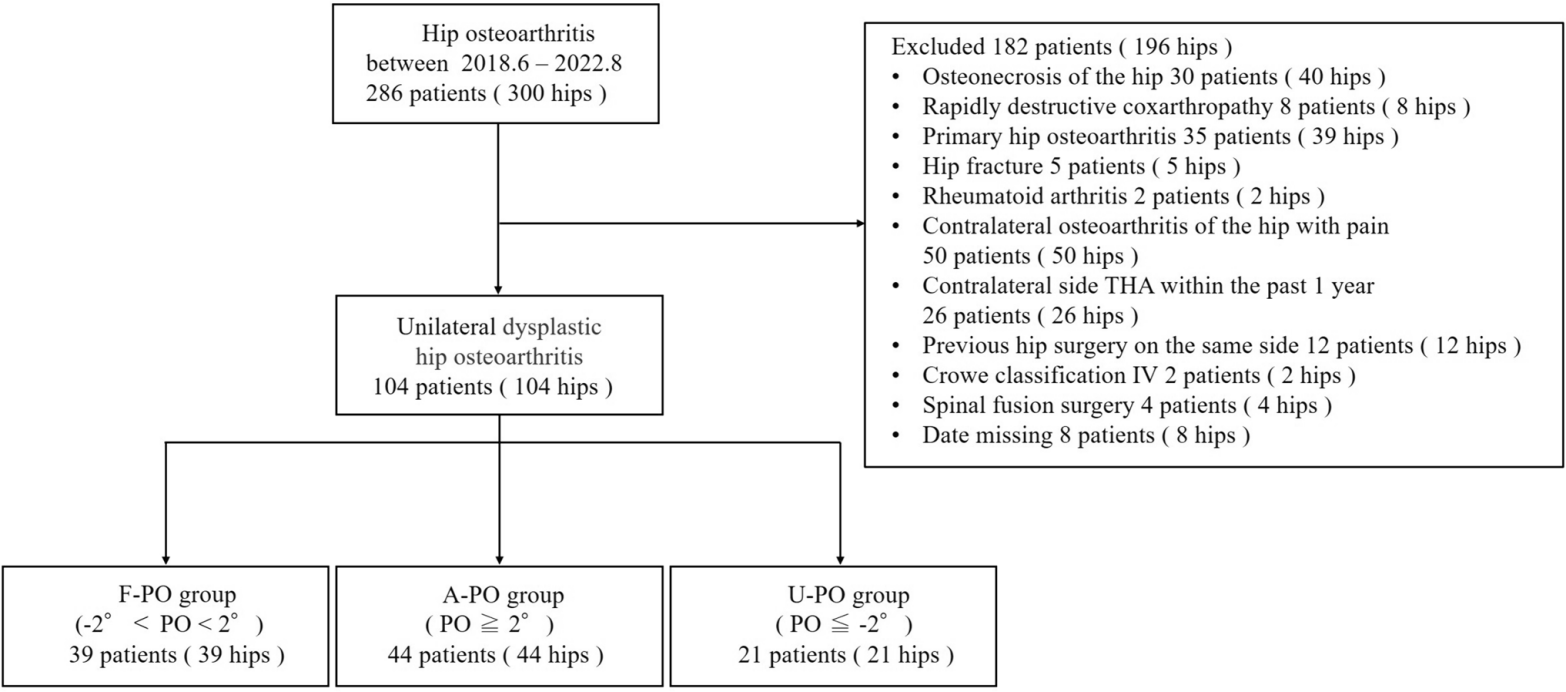
Inclusion criteria for this study. Patients with − 2° < PO < 2° are categorized into the flat (F-PO) group. Patients with PO ≥ 2° are categorized into the affected side (A-PO) group. Patients with PO ≤ − 2° are categorized into the unaffected side (U-PO) group

This study was performed under the certification of the Ethics Committee of Nagoya University Hospital. This retrospective cohort study examined 286 patients (300 hips) who referred at our hospital between June 2018 and August 2022. From these, 104 patients (104 hips) who had unilateral osteoarthritis secondary to developmental dysplasia of the hip (DDH) were included. The remaining 196 hips in patients who had osteonecrosis of the femoral head (n = 40), rapidly destructive hip joint disease (n = 8), primary hip joint disease (n = 39), fractures (n = 5), rheumatoid arthritis (n = 2), contralateral hip osteoarthritis with pain (n = 50), contralateral total hip arthroplasty within 1 year (n = 26), a history of hip surgery (n = 12), missing data (n = 8), Crowe type IV dysplasia (n = 2), and a history of spinal fixation surgery (n = 4) were excluded. According to previous reports, the average lateral PO in healthy individuals is approximately 2° [7]. Therefore, we conducted a case–control study, categorizing patients based on standing anteroposterior hip radiographs into three groups: the flat group with PO < 2° (F-PO group), affected side group with the affected side PO downward by ≥ 2° (A-PO group), and unaffected side group with the unaffected side PO downward by ≥ 2° (U-PO group) (Fig. 1).

### Hip and lower limb radiographic evaluation

**Fig. 2:**
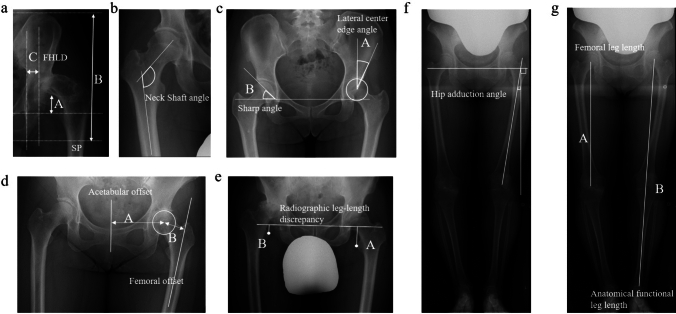
Radiographic evaluation of the lower limbs. **a** Subluxation percentage: 5A/B, Femoral head lateralization distance: C. SP: Subluxation percentage, FHLD: Femoral head lateralization distance. **b** Neck shaft angle. **c** Lateral center edge angle: A, sharp angle: B. **d** Femoral offset: A, acetabular offset: B. **e** Radiographic leg-length discrepancy: A-B. **f** Hip adduction angle. **g** Femoral leg length: A, Anatomical functional leg length: B

Radiographic evaluation of the hip and lower limbs included the subluxation percentage, Crowe classification, femoral head lateralization distance, neck shaft angle, lateral center edge angle, sharp angle, acetabular offset discrepancy, femoral offset discrepancy, radiographic leg-length discrepancy (R-LLD), hip adduction angle, anatomical femoral length discrepancy, and functional leg-length discrepancy [8]. Standing anteroposterior hip or full-length lower extremity radiographs were used for the evaluation. Following the Crowe classification, the hip subluxation percentage was determined and each hip was categorized into groups ranging from I to IV [9, 10]. The distance between the medial aspect of the femoral head and ilioischial line was defined as the femoral head lateralization distance [11]. The neck shaft, lateral center edge, and sharp angles were measured, as previously reported [12, 13]. The distance between the center of the femoral head and the perpendicular line passing through the pubic symphysis was defined as the acetabular offset. Femoral offset was defined as the distance between the longitudinal axis of the femur and the center of the femoral head [14]. R-LLDs were measured using the method proposed by Woolson and Harris [15]. A pelvic reference line passing through the lowest points of the bilateral acetabular rims was established. Two lines parallel to the inter-teardrop line were drawn through the center of the lesser trochanters. The disparity in the perpendicular distance between the unaffected and affected sides was considered R-LLD, with a positive value indicating shortening of the hip joint on the affected side [15]. Hip adduction angle was determined by measuring the angle between the perpendicular line extending from the lower tip of the bilateral pelvic teardrops and the longitudinal axis of the femur on the affected side [16]. Anatomical femoral length was defined as the distance between the centers of the femoral head and trochlea. Functional leg length was defined as the distance between the centers of the femoral head and ankle joint [3]. The acetabular and femoral offset discrepancies were defined as the values obtained by subtracting the values of the unaffected side from those of the affected side. The hip-adduction angle was considered positive in the inward direction. For functional leg-length and anatomical femoral length discrepancies, the values were determined by subtracting the measurements on the unaffected side from those on the affected side (Fig. 2).

Thirty cases were evaluated for radiological measurements by two surgeons. The inter-observer reliability values for various parameters were assessed as follows: subluxation percentage (0.82), femoral head lateralization distance (0.84), neck shaft angle (0.82), lateral center edge angle (0.88), sharp angle (0.92), acetabular offset discrepancy (0.78), femoral offset discrepancy (0.80), R-LLD (0.82), hip adduction angle (0.86), anatomical femoral length discrepancy (0.89), and functional leg-length discrepancy (0.87).

### Spinal radiographic evaluation

**Fig. 3:**
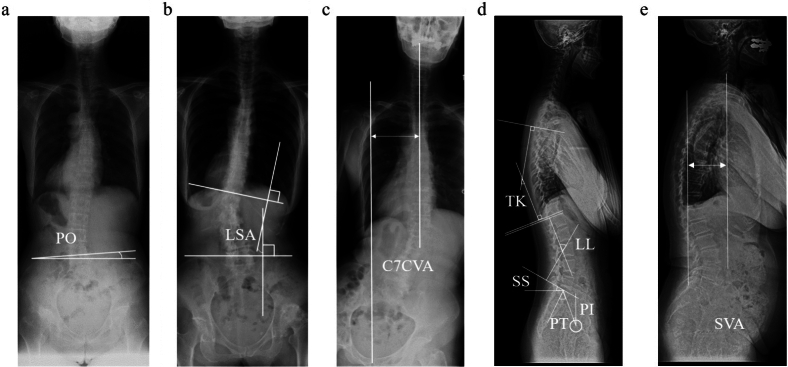
Radiographic evaluation of spinal parameters. **a** Pelvic obliquity **b** Lumbar scoliosis angle **c** C7 coronal vertical axis **d** TK: thoracic kyphosis, LL: lumbar lordosis, SS: sacral slope, PI: pelvic incidence, and PT: pelvic tilt **e** SVA: sagittal vertical axis

At the time of the initial consultation, we conducted a radiographic assessment of the spine and pelvis, focusing on preoperative factors such as PO, C7 coronal vertical axis (C7CVA), lumbar scoliosis angle, and sagittal parameters. During this evaluation, we analyzed the anteroposterior and lateral radiographs of the patients in a standing position. PO was determined by measuring the angle between the horizontal line tangent to the most proximal iliac crest, following the methodology described by Osebold et al. [17]. PO, where the affected side tilts downward, is represented as a positive value, whereas PO with the affected side tilting upward is represented as a negative value. The C7CVA and lumbar scoliosis angle were measured according to the method of Nakashima et al. [18]. The C7CVA was defined as the horizontal distance from the center sacral vertical line to C7, with the affected side considered as the positive direction. The lumbar scoliosis angle was defined as the angle between the line extending from the superior border of the L1 lumbar vertebra and the line passing through the superior border of both iliac crests. A positive value indicated that the lumbar scoliosis angle was convex to the hip joint on the affected side. Sagittal plane parameters were investigated using standing full-spine radiographs, including pelvic tilt (PT), sacral slope (SS), pelvic incidence (PI), lumbar lordosis (LL), thoracic kyphosis (TK), and C7 sagittal vertical axis (SVA). These measurements were taken as previously described [17, 18]. C7SVA was defined as the distance from the central tangent of C7 to the upper sacral end (a positive value was defined when C7 was further forward) (Fig. 3).

Thirty cases underwent radiological measurements conducted by two surgeons, and their inter-observer reliability values were evaluated. The inter-observer reliability values for various parameters were as follows: PO (0.80), C7CVA (0.78), lumbar scoliosis angle (0.81), PT (0.79), SS (0.83), PI (0.85), LL (0.80), TK (0.86), and C7SVA (0.81).

### Clinical evaluation

Demographic information, including sex, age, height, weight, body mass index (BMI), and status of the contralateral hip, was retrieved from the patients' medical records.

The clinical evaluation included the Harris hip score (HHS) and ROM. Hip function was assessed using the HHS and ROM at the time of the initial consultation. The ROM was measured by a senior surgeon using a goniometer. The HHS was measured by a senior surgeon.

### Data analyses

We used EZR software (Saitama Medical Center, Jichi Medical University) for all statistical analyses [19]. One-way analysis of variance was employed for continuous variables, followed by the Bonferroni post hoc test. Fisher’s exact test was used for categorical variables, with the Bonferroni test as a post hoc test. The significance level was set at P < 0.05. Age, sex, and BMI were included as factors in the multivariate logistic regression analysis, where P < 0.05 was considered significant. Inter-observer reliability was evaluated using intraclass correlation coefficients (ICCs), with one indicating perfect correlation and zero indicating poor correlation. The measurements were performed by two surgeons.

## Results

There were 39, 44, and 21 patients in the F-PO, A-PO, and U-PO group, respectively. Sex, age, weight, and BMI did not differ significantly among the three groups. The height of patients in the F-PO group (158.9 ± 9.3 cm) was significantly greater than that in the A-PO group (154.4 ± 7.5 cm; p = 0.024) (Table 1).

**Table 1:** Patients demographics

	F-PO group (n = 39)	A-PO group (n = 44)	U-PO group (n = 21)	p
Sex (male/female)	12/27	6/38	3/18	0.152
Age (y)	63.4 ± 9.3	66.7 ± 10.2	64.5 ± 12.2	0.216
Height (cm)	158.9 ± 9.3	154.4 ± 7.5	154.9 ± 5.9	0.024^a^
Weight (kg)	61.5 ± 12.3	57.0 ± 10.8	59.8 ± 13.2	0.216
Body mass index (kg/m^2^)	24.3 ± 4.0	23.9 ± 3.8	25.0 ± 5.6	0.598
Other side (Healthy / THA)	36 / 3	36 / 8	14/7	0.049

THA: Total hip arthroplasty

^a^F-PO group vs A-PO group: p < .05

**Fig. 4:**
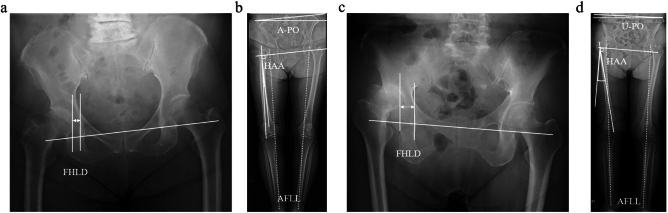
Representative cases. **a**, **b** A case with A-PO. Minimal lateral and superior displacement of the femoral head, along with abduction of the hip joint, resulting in a shortened functional leg length on the affected side. **c**, **d** A case with U-PO. Lateral and superior displacement of the femoral head, with adduction of the hip joint. FHLD: Femoral head lateralization distance, A-PO: affected side pelvic obliquity, U-PO: Unaffected side pelvic obliquity, HAA: Hip adduction angle, AFLL: Anatomical functional leg length

The subluxation percentage showed a significant difference among the three groups, with values of 9.0 ± 12.7, 20.5 ± 19.5, and 34.8 ± 17.7 in the F-PO, A-PO, and U-PO groups, respectively (p < 0.001). The femoral head lateralization distance was significantly greater in the U-PO group (21.0 ± 5.6 mm) than in the F-PO (15.9 ± 4.6 mm) and A-PO (16.1 ± 4.7 mm) groups, indicating a large lateralization in the U-PO group (p < 0.001). Furthermore, the hip adduction angle was significantly lower in the A-PO group (2.2 ± 3.9°) than in the F-PO (6.1 ± 4.0°) and U-PO (8.9 ± 5.8°) groups, indicating a tendency to exhibit hip abduction in the A-PO group (p < 0.001). The R-LLD in the F-PO group (6.1 ± 6.8 mm) was significantly smaller than that in the A-PO (11.9 ± 11.0 mm) and U-PO (16.4 ± 8.8 mm) groups (p < 0.001). The functional leg-length discrepancies were significantly different in the F-PO (− 1.4 ± 5.4 mm), A-PO (3.1 ± 8.4 mm), and U-PO (− 6.9 ± 14.0 mm) groups (p < 0.001) (Table 2, Fig. 4).

**Table 2:** Radiographic evaluation of the lower limbs

	F-PO group (n = 39)	A-PO group (n = 44)	U-PO group (n = 21)	p
Subluxation percentage	9.7 ± 14.4	20.3 ± 21.6	38.2 ± 24.3	< 0.001^abc^
Crowe classification (I/II/III)	38/1/0	39/4/1	15/5/1	0.025^c^
Femoral head lateralization distance (mm)	15.9 ± 4.6	16.1 ± 4.7	21.0 ± 5.6	< 0.001^bc^
Neck shaft angle (degree)	134.8 ± 8.4	135.1 ± 9.4	137.8 ± 12.3	0.484
Lateral center edge angle (degree)	13.1 ± 10.9	12.5 ± 10.6	11.2 ± 9.8	0.796
Sharp angle (degree)	45.9 ± 6.0	45.0 ± 6.0	44.0 ± 4.8	0.492
Acetabular offset discrepancy (mm)	− 3.2 ± 10.0	− 4.8 ± 9.8	− 1.5 ± 13.9	0.502
Femoral offset discrepancy (mm)	4.4 ± 7.5	4.7 ± 8.5	0.0 ± 10.7	0.104
Radiographic leg length discrepancy (mm)	6.1 ± 6.8	11.9 ± 11.0	16.4 ± 8.8	0.001^abc^
Hip adduction angle (degree)	6.7 ± 3.8	2.2 ± 3.9	8.9 ± 5.8	< 0.001^abc^
Anatomical femoral length discrepancy (mm)	− 0.9 ± 4.2	2.7 ± 7.5	− 0.1 ± 10.0	0.056
Functional leg length discrepancy (mm)	− 1.4 ± 5.4	3.1 ± 8.4	− 6.9 ± 14.0	< 0.001^b^

^a^F-PO group vs A-PO group: p < .05. ^b^A-PO group vs U-PO group: p < .05. ^c^F-PO group vs U-PO group: p < .05

The lumbar scoliosis angle was significantly different in the F-PO (0.9 ± 4.8°), A-PO (− 2.7 ± 10.1°), and U-PO (1.6 ± 6.3°) groups (p = 0.038). The lumbar scoliosis of group A exhibited convexity toward the healthy side compared to that of group H. However, there were no significant differences in sagittal spinal parameters among the three groups (Table 3).

**Table 3:** Radiographic evaluation of the spinal alignment

	F-PO group (n = 39)	A-PO group (n = 44)	U-PO group (n = 21)	p
Pelvic obliquity (degree)	0.3 ± 0.9	4.0 ± 1.8	− 4.3 ± 1.2	0.001^abc^
Lumbar scoliosis angle (degree)	0.9 ± 4.8	− 2.7 ± 10.1	1.6 ± 6.3	0.038^b^
C7CVA (mm)	10.1 ± 18.0	13.6 ± 13.1	9.5 ± 21.4	0.533
LBR (degree)	8.9 ± 5.2	8.8 ± 5.6	8.0 ± 5.8	0.823
PT (degree)	14.3 ± 14.7	17.2 ± 9.3	15.5 ± 11.7	0.267
SS (degree)	34.2 ± 10.4	35.5 ± 10.9	33.5 ± 7.8	0.729
PI (degree)	49.8 ± 10.2	52.6 ± 9.8	48.9 ± 9.3	0.267
LL (degree)	39.0 ± 14.1	37.2 ± 13.4	37.6 ± 12.6	0.832
PI-LL (degree)	9.6 ± 17.3	15.4 ± 14.1	11.3 ± 16.1	0.231
TK (degree)	26.9 ± 11.4	24.3 ± 12.6	26.7 ± 12.9	0.564
SVA (mm)	28.0 ± 34.5	33.6 ± 38.8	36.5 ± 24.5	0.621

C7CVA: C7 coronal vertical axis, LBR: Lumber bending range, PT: pelvic tilt, SS: sacral slope, PI: pelvic incidence, LL: lumbar lordosis, TK: thoracic kyphosis, SVA: sagittal vertical axis

^a^F-PO group vs A-PO group: p < .05. ^b^A-PO group vs U-PO group: p < .05. ^c^F-PO group vs U-PO group: p < .05

In multivariate analysis, hip adduction angle (OR, 0.742; 95% CI 0.627–0.880; p < 0.01) was extracted as an independent factor associated with A-PO (Table 4). In contrast, age (OR, 1.110; 95% CI 1.000–1.220; p = 0.043), subluxation percentage (OR, 1.060; 95% CI 1.000–1.120; p = 0.035), and hip adduction angle (OR, 1.470; 95% CI 1.100–1.970; p = 0.009) were identified as independent factors associated with U-PO (Table 5).

**Table 4:** Results of multivariate analysis; Logistic regression analysis A-PO group

	B	OR (95% CI)	p
Age (y)	0.015	1.020 (0.954–1.080)	0.636
BMI	− 0.050	0.951 (0.813–1.110)	0.530
Sex (male)	− 1.295	0.274 (0.064–1.170)	0.081
Subluxation percentage	0.013	1.010 (0.974–1.050	0.522
Radiographic leg length discrepancy (mm)	0.046	1.590 (0.964–1.140)	0.274
Hip adduction angle (degree)	− 0.298	0.742 (0.627–0.880)	< 0.001

BMI: body mass index

**Table 5:** Results of multivariate analysis; Logistic regression analysis U-PO group

	B	OR (95% CI)	p
Age (y)	0.101	1.110 (1.000–1.220)	0.043
BMI	0.078	1.080 (0.882–1.330)	0.450
Sex (male)	− 0.014	0.986 (0.087–0.991)	0.991
Subluxation percentage	0.058	1.060 (1.000–1.120)	0.035
Radiographic leg length discrepancy (mm)	0.131	1.140 (0.992–1.310)	0.065
Hip adduction angle (degree)	0.385	1.470 (1.100–1.970)	0.009
Femoral head lateralization distance (mm)	0.116	1.120 (0.910–1.380)	0.279

BMI: body mass index

The HHS total was significantly poorer in U-PO group (53.0 ± 14.8) than in F-PO group (62.3 ± 14.0) (p = 0.046). The ROM in flexion and extension was significantly smaller in U-PO group (79.5 ± 25.1° and − 0.2 ± 9.4°) than in F-PO group (95.8 ± 19.3° and 7.0 ± 8.0°) (p = 0.009 and 0.008) (Table 6).

**Table 6:** Clinical evaluations

	F-PO group (n = 39)	A-PO group (n = 44)	U-PO group (n = 21)	p
Harris hip score				
Pain	21.3 ± 8.1	19.1 ± 8.4	20.5 ± 8.6	0.477
Function	32.2 ± 6.2	29.7 ± 10.0	25.6 ± 8.9	0.021^c^
Total	62.3 ± 14.0	56.4 ± 15.1	53.0 ± 14.8	0.046^c^
Range of motion				
Flexion	95.8 ± 18.9	89.3 ± 15.8	79.5 ± 25.1	0.009^c^
Extension	7.0 ± 8.0	2.7 ± 9.2	− 0.2 ± 9.4	0.008^c^
Abduction	21.2 ± 9.1	20.1 ± 10.5	15.5 ± 10.5	0.105
Adduction	15.4 ± 12.5	14.8 ± 8.6	12.6 ± 9.0	0.602
External rotation	24.2 ± 10.9	19.3 ± 12.5	18.1 ± 16.2	0.216
Internal rotation	17.4 ± 14.4	18.8 ± 15.3	9.8 ± 11.5	0.056

^c^F-PO group vs U-PO group: p < .05

## Discussion

In this study, we examined the PO in patients who have DHOA, focusing on morphological characteristics, spinal alignment factors, and clinical outcomes. The morphological characteristic of the A-PO in DHOA was identified as the hip adduction angle. The morphological characteristics of the U-PO in DHOA were age, subluxation percentage, and hip adduction angle. The only factor associated with the direction of PO in patients who have unilateral DHOA was the lumbar scoliosis angle. Patients who have U-PO in DHOA had poorer HHS and hip ROM.

The cause of A-PO in DHOA is considered to be compensation for the short leg on the affected side. In patients who have polio, A-PO is evident in cases involving both the shorter sides of the lower limb and instances of external rotation contracture [5]. In patients who have leg-length discrepancies, a compensatory mechanism during walking involves tilting the pelvis downward on the side of the shortened leg, which induces an abducted position at the hip joint [20]. In this study, patients who have A-PO exhibited a significantly shortened functional leg length. The hip adduction angle in the standing position was significantly smaller, with a relative femoral abduction. Furthermore, hip adduction angle was identified as an independently associated factor. Shortening of the affected limb compared to the unaffected side is considered a contributing factor to A-PO, compensating for alignment by exhibiting hip abduction. These cases were considered prevalent in patients who have mild subluxation in DHOA without accompanying hip adduction contractures.

The factors leading to U-PO in patients who have DHOA remain unclear. In particular, despite the presence of a radiographic leg-length discrepancy, the occurrence of U-PO suggests the possibility of further leg-length discrepancies. In patients who have polio, hip adduction contracture has been reported to shorten the leg side to induce an upward PO [5]. A decrease in abductor muscle strength is also considered a factor causing U-PO [21]. In this study, the patients who have U-PO exhibited a significantly larger percentage of subluxation, femoral head lateralization distance, and hip adduction angles. In contrast, functional leg-length discrepancy was significantly greater on the affected side than on the unaffected side. Age, subluxation percentage, and hip adduction angle were identified as independent factors. Tani et al. reported a significant association between severe adduction contracture and severity of subluxation in cases of hip osteoarthritis [22]. In severe DHOA, it is presumed that U-PO compensates for functionally lengthened legs due to adduction contracture. Age may have been an independent factor considering that the degree of subluxation tends to progress with increasing age.

Scoliosis and pelvic tilt are strongly correlated [23]. In DHOA, spinal flexibility has been shown to influence the PO. However, the relationship between spinal parameters and PO in patients who have DHOA has not been clearly established. In this study, a larger lumbar scoliosis angle was observed in patients who have an A-PO. To maintain balance in the coronal plane of the spine, the lumbar scoliosis angle on the affected side was compensated for by becoming concave. However, no significant factors were observed for the other spinal parameters. In scoliosis, various spinal parameters have been shown to be associated with the PO [21]. Since this study specifically focused on PO in DDH, and cases with significant scoliosis were limited, this may not be fully reflected in the results.

It is not clear whether the direction of the PO affects the hip joint function in DHOA. PO was associated with perceived leg-length discrepancies and external rotator muscle strength in patients who have hip osteoarthritis [7, 21]. In this study, the U-PO group exhibited impaired flexion and extension with a tendency towards poorer HHS. In patients who have U-PO, the subluxation percentage was high, suggesting that the progression of osteoarthritis changes strongly influences hip joint function. Early surgical intervention is recommended in patients who have U-PO.

This study had several limitations. It was a retrospective cohort study. Assessing changes in the PO in DHOA over time will be a topic for future research. The small number of cases was another limitation. U-PO was more common in patients who have severe subluxation in DHOA. Severe hip subluxation is rare in DHOA, and only 21 of 104 patients had upward PO. However, few studies have evaluated factors associated with PO in patients who have DHOA. Additionally, slight variations in radiographic measurements may result from differences in patient positioning during scanning. Accurate alignment for X-rays cannot always be ensured, potentially causing minor measurement inaccuracies.

In conclusion, the hip adduction angle was an independent factor associated with the A-PO. Age, subluxation percentage, and hip adduction angle were independent factors associated with the U-PO. The lumbar scoliosis angle was only associated with the PO direction. Furthermore, patients who have U-PO exhibited significantly poorer HHS than those who have A-PO.

## Data Availability

The datasets generated and/or analyzed during the current study are available from the corresponding author on reasonable request.
